# Vibration Rather than Microgravity Affects Bone Metabolism in Adult Zebrafish Scale Model

**DOI:** 10.3390/cells13060509

**Published:** 2024-03-14

**Authors:** Marta Carnovali, Stefania Zava, Giuseppe Banfi, Angela Maria Rizzo, Massimo Mariotti

**Affiliations:** 1 IRCCS Ospedale Galeazzi Sant’Ambrogio, Via C. Belgioioso 173, 20161 Milan, Italy; marta.carnovali@grupposandonato.it (M.C.); giuseppe.banfi@hsr.it (G.B.); 2 Department of Pharmacological and Biomedical Sciences “Rodolfo Paoletti”, University of Milan, Via D. Trentacoste 2, 20134 Milan, Italy; stefania.zava@unimi.it (S.Z.); angelamaria.rizzo@unimi.it (A.M.R.); 3 School of Medicine, Vita-Salute San Raffaele University, Via Olgettina 58, 20132 Milan, Italy; 4 Department of Biomedical, Surgical and Dental Sciences, University of Milan, Via Commenda 10, 20122 Milan, Italy

**Keywords:** zebrafish, microgravity, scale, bone

## Abstract

Gravity and mechanical forces cause important alterations in the human skeletal system, as demonstrated by space flights. Innovative animal models like zebrafish embryos and medaka have been introduced to study bone response in ground-based microgravity simulators. We used, for the first time, adult zebrafish in simulated microgravity, with a random positioning machine (RPM) to study bone remodeling in the scales. To evaluate the effects of microgravity on bone remodeling in adult bone tissue, we exposed adult zebrafish to microgravity for 14 days using RPM and we evaluated bone remodeling on explanted scales. Our data highlight bone resorption in scales in simulated microgravity fish but also in the fish exposed, in normal gravity, to the vibrations produced by the RPM. The osteoclast activation in both rotating and non-rotating samples suggest that prolonged vibrations exposure leads to bone resorption in the scales tissue. Stress levels in these fish were normal, as demonstrated by blood cortisol quantification. In conclusion, vibrational mechanical stress induced bone resorption in adult fish scales. Moreover, adult fish as an animal model for microgravity studies remains controversial since fish usually live in weightless conditions because of the buoyant force from water and do not constantly need to support their bodies against gravity.

## 1. Introduction

Gravity plays a primary role in human physiology. Indeed, the lack of gravity, like during spaceflight missions, causes important alterations in the skeletal system [1].

In the absence of real space flight, to facilitate gravity-related studies about the effects on biological systems, ground-based microgravity simulators represent an important resource [2].

Recently, 3D in vitro models have been proposed to study the molecular mechanisms of tissue regulation in low gravity [3], with appropriate limitations. Regarding in vivo animal models, rodents have been often used in ground experiments. In particular, the mouse tail suspension method represents an elective technique to simulate microgravity, given its simplicity and scalability [4]. Nevertheless, such simulation is still quite far from the real gravitational unloading found in space. In fact, suspended rodents have also shown additional dysfunctions with respect to spaceflight conditions, like spine deformity [5] or aging-like neurophysiological change [6].

Previous studies have demonstrated that fishes, even though they are water-living animals, are sensitive to microgravity, which influence swimming behavior, growth, metabolism and bone formation in larvae [7,8,9,10,11].

Indeed, the high similarity of the fish skeletal system with humans makes this animal model suitable for gravity studies independently of their habitat. In fact, Danio rerio (zebrafish) and medaka have been introduced to study the regulatory pathways of bone homeostasis involved in skeletal disorders caused by altered gravity in higher vertebrates [12].

Previous experiments with increased mechanical pressure on zebrafish embryos have been performed by Aceto et al. [11], in which 5dpf embryo were treated with hypergravity (3G) using a Large Diameter Centrifuge (LDC) for up to 9 days post fertilization (dpf). Similar experiments were performed by Lawrence et al. that exposed 3dpf embryos at 3G and 6G for 48 h. Lawrence’s study reported that embryo morphology is unchanged after a short hypergravity treatment, but it evidenced some changes in the cartilaginous matrix and altered chondrocyte maturation [13].

On the other hand, a reduction in gravity, generated by a 2D clinorotation for 5 days, caused a significant decrease of skeletal tissue formation, both cartilage and bone, in zebrafish embryos. This result was due to a modulation of musculoskeletal gene expression rather than the role of stress, measured as cortisol levels [14].

More recently, it was highlighted that both vibration and simulated microgravity affect osteogenesis in zebrafish larvae in terms of delayed ossification, and a reduced vertebrae number and body length [15].

Since bone formation delay during zebrafish embryo development differs from adult osteoporosis observed in astronauts, we introduced the in vivo scale model to investigate whether a bone-loss phenotype is promoted in adult skeletal tissue in the microgravity condition.

In addition, during space colonization, animals should be able to be brought. Our study on adult fish skeletal behavior in simulated microgravity conditions could also be relevant to set up the management of animals during long space flight.

The use of a scale model in adult zebrafish represents an excellent read-out system to investigate the modulation of bone metabolism after physical stimulation like microgravity [16].

In this work, we analyzed a mineralized matrix in adult zebrafish scale after treatment with simulated microgravity, generated by a random positioning machine (RPM). 

## 2. Materials and Methods

### 2.1. Animals

Nine-month-old male *Danio rerio* of an AB strain, of comparable lengths and sizes, were housed in a ZEBTEC© Bench Top System (Tecniplast, Buguggiate VA, Italy) and maintained under standard conditions [17] at 28 °C. 

### 2.2. Ethic Statement

This experiment was performed in the Zebrafish Laboratory (IRCCS R. Galeazzi, GSD Foundation, Milan, Italy) according to the Italian and European guidelines on research practice (EU Directive 2010/63/EU), with the approval of Italian Ministry of Health (authorization n. 1028/2020-PR).

### 2.3. Microgravity Treatment

**Figure 1:**
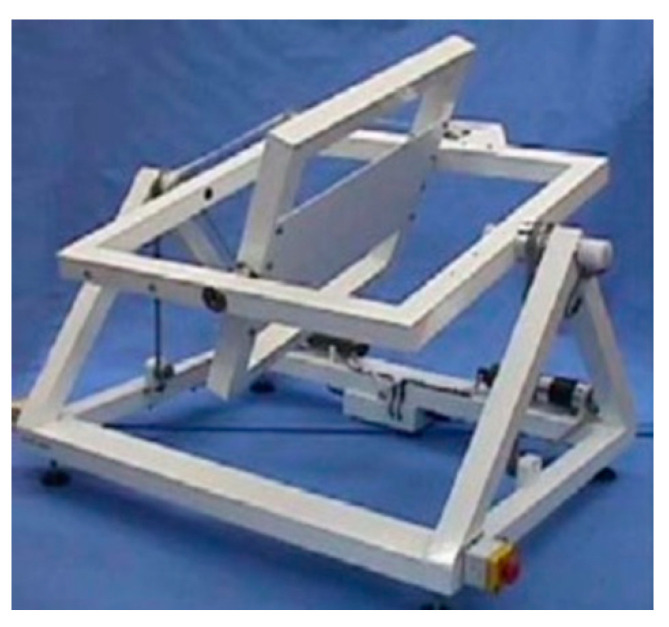
The full-size Random Positioning Machine (RPM); credit Dutch Space. The experiment was fixed in the center of the inner platform during random rotation.

Simulated microgravity was obtained using a Random Positioning Machine (RPM, Dutch Space, Leiden, The Netherlands); the RPM is a commercial tool that consists of two independently rotating frames, positioned one inside the other, randomly rotated with different directions, velocities and angles. A classical 3D clinostat rotates both axes in the same direction (i.e., both clockwise). The RPM is a simulator that is based on the principle of ‘gravity-vector-averaging’. The device was set up in 3D random mode, with the samples housed in the center of the platform in the inner ring, as previously described by van Loon et al. [18,19], to reach 10^−3^× *g*, allocated in a temperature-controlled room [18,19] (Figure 1).

Fish treatments were performed in E3 medium (5 mM NaCl, 0.17 mM KCl, 0.33 mM CaCl_2_, 0.33 mM MgSO_4_) [13], with a single fish housed in a homemade fish microgravity box adapted for simulation experiments, as fully described in the results section. Briefly, we used a commercial food-grade polyethylene square box (10 cm × 10 cm × 4 cm) and a gas-permeable (CO_2_/O_2_) fluorocarbon foil 25 μm thick transparent film (Lumox^TM^ film, Sarstedt, Nümbrecht, Germany). The experimentation included a control group (CTR), with fish simply maintained on a laboratory table in the microgravity box, the rotating group (R) maintained in constant rotation for 14 days, and a non-rotating group (NR), an additional control positioned on the non-rotating base of the instrument, constantly exposed to vibrations but not to microgravity. Every 24 h, the fish were fed and the E3 medium entirely changed. During this pause, we checked fish behavior, looking for alterations in swimming ability and food intake. Temperature, humidity and a 14:10 light/dark cycle in the room were all controlled to guarantee fish behavior. 

### 2.4. Samples Collection

At the end of the treatment, fish were anaesthetized in 0.01% tricaine methanesulfonate (MS222, Sigma, St. Louis, MO, USA) E3 medium solution and their scales were carefully removed from either fish body side using Dumont^®^ stainless steel forceps (Sigma Aldrich, St. Louis, MO, USA) under light stereomicroscope (Olympus SZX-ZB7, Tokyo, Japan). Scales were fixed in 3.5% formaldehyde 0.1 M sodium phosphate-buffered solution (pH 7.4), repeatedly washed in phosphate-buffered saline (PBS) and processed differently, as described below, depending on the analysis to perform. Then, fish blood was collected according to previously published protocol [20]. 

### 2.5. Bone Matrix Staining

Scales were stained using 0.005% Calcein (Bis[*N*,*N*−bis(carboxymethyl)aminomethyl] fluorescein, Sigma Aldrich, St. Louis, MO, USA) E3 staining solution for 30 min in the dark. After repeated washes in E3 medium solution, scales were analyzed using a fluorescence microscope (Olympus SZX-ZB7, Tokyo, Japan) equipped with a Discovery CH30 camera (TiEsseLab, Milan, Italy). The analysis of imagines with ISC Capture Software 3.6 allowed to quantify the mineralized matrix. 

### 2.6. Histological and Biochemical TRAP Activity

Histological tartrate-resistant acid phosphatase (TRAP) activity was evaluated using Leukocytes Acid Phosphatase (TRAP) Detection Kit (Sigma Aldrich, St. Louis, MO, USA), processing the scales according to manufacturer’s protocol. Images of stained scales were collected using a light stereomicroscope (Olympus SZX-ZB7, Tokyo, Japan). Biochemical TRAP activity was evaluated on explanted scales following a previously published method [21].

### 2.7. Cortisol Quantification

Fish blood from 3 fish of each treatment group was pooled and used for Cortisol quantification by ELISA testing using a Fish Cortisol ELISA Kit (MyBioSource, San Diego, CA, USA), according to manufacturer’s protocol. Absorbance of the resulting test solutions were read at 450 nm using a spectrophotometer (iMarkTM Microplate Reader, Bio-Rad, Hercules, CA, USA).

### 2.8. Statistics

Each experiment consisted of treatment for 14 days of one fish for each group, control (CTR), clinostat rotating (R) and clinostat non-rotating (NR), which were singularly located in the boxes. Three independent experiments were conducted for a total of three animals per group. In each experiment, histological and biochemical analyses were performed on 30 scales for each fish, while the blood samples of the 3 fish of each treatment group were pooled to perform cortisol blood analysis. Statistical significance was determined for *p*-values by using ANOVA followed by the Bonferroni test for multiple comparisons. All the significance values were set at *p* < 0.05 (*), *p* < 0.01 (**) and *p* < 0.001 (***).

## 3. Results 

### 3.1. Microgravity Fish-Box

**Figure 2:**
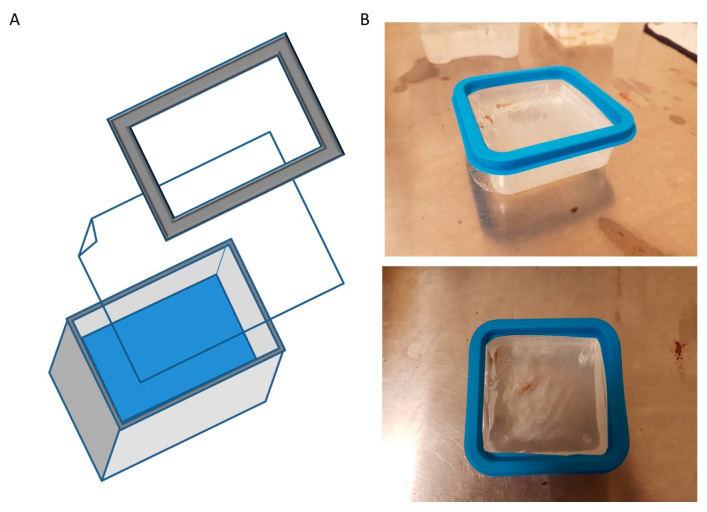
Fish microgravity box. (**A**) A commercial food-grade polyethylene square box (10 cm × 10 cm × 4 cm) was used adding a gas permeable transparent film on top, sealed with cut cover. ((**B**) **upper** and **lower**). The box was filled with E3 medium and used during the experiment to house a single adult fish.

A real microgravity condition can be reached in an RPM device following precise physical parameters and rules; in particular, to achieve a gravity vector of about 10^−3^× *g,* the most important condition is to place the experimental setup in the center of the rotating platform, shown in Figure 1 [18]. In the in vivo experiments, the animal should be allocated in an appropriate container that guarantees animal well-being and housing on the RPM, without leaking during of medium during the rotation. Since containers of this type are not commercially available, a homemade box (Fish Microgravity Box) was prepared to host one adult zebrafish for the entire course of the experiment. A commercial food-grade polyethylene square box (10 cm × 10 cm × 4 cm) was rinsed with 300 mL of fish water and an adult fish was placed inside. Then, a gas-permeable (CO_2_/O_2_) fluorocarbon foil 25 μm thick transparent film (see material and method section) was laid flat to cover the box, in contact with the water, to avoid air bubbles during rotation. The inner part of the box cover was cut away, leaving only the cornice used to seal the paper to the box (Figure 2A,B).

Preliminary tests indicated that the system was able to guarantee animal wellbeing during a long time of housing.

### 3.2. Microgravity Simulation Was Associated to Mineral Matrix Loss in Zebrafish Scale

In order to test whether microgravity can affect the bone tissue remodeling, adult zebrafish were placed in the box at the center of the RPM plate and left in rotation for 14 days (R). An untreated control was prepared in the same type of box and located in a separated table in the same room (CTR). Since the clinostat produces vibrations during the rotation activity, an additional control was positioned on the non-rotating basement of the instrument (NR). Every 24 h, the fish were fed, and the water entirely changed; during the pause, the analysis of fish behavior (i.e., swimming ability and food intake) indicated a rapid adaptation to microgravity simulation. All the fish survived the three repeated experiments. 

**Figure 3:**
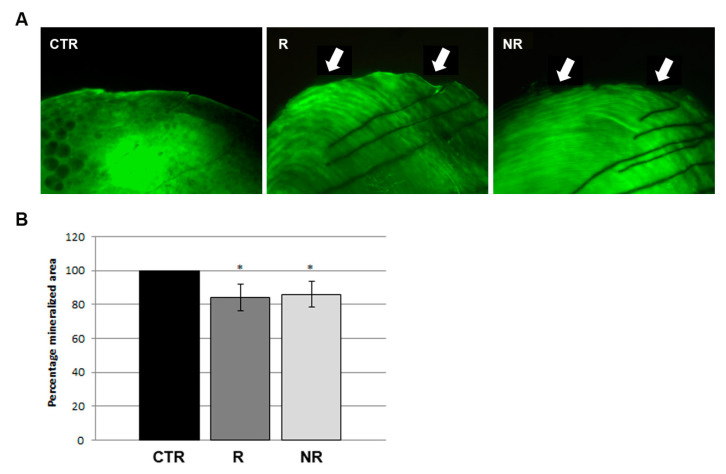
Evaluation of scale mineralization. (**A**) Calcein staining of scales. Unlike the controls (CTR), both scales from rotating (R) and non-rotating (NR) fish showed discontinuity in a mineralized matrix along the borders (white arrows). (**B**) Quantification of scale area showed a reduction of 15% in the clinostat-treated samples (R and NR) with respect to the untreated control fish (CTR). (R vs. CTR, −15.8%, * *p* < 0.05; NR vs. CTR, −13.9%, * *p* < 0.05, *n* = 90 scales in each group).

At the end of the experiment, we performed a mineralized matrix vital staining with calcein on the controls and the rotating and non-rotating fish. Scales of control fish displayed a perfectly formed scale border; on the contrary, 100% scales of RPM rotating fish showed altered morphology and extended bone loss along the anterior border. Interestingly, a similar bone-loss phenotype was found in the non-rotating fish (Figure 3A). The quantification of the scale-mineralized area confirmed the bone resorption in rotating (−15.8%) and non-rotating (−13.9%) fish with respect to the untreated controls. (Figure 3B). These data suggest that both simulated microgravity and RPM vibrations might modulate bone remodeling in fish scales.

### 3.3. Bone Resorption Was Promoted by Osteoclast Activity in Scales after Simulated Microgravity

**Figure 4:**
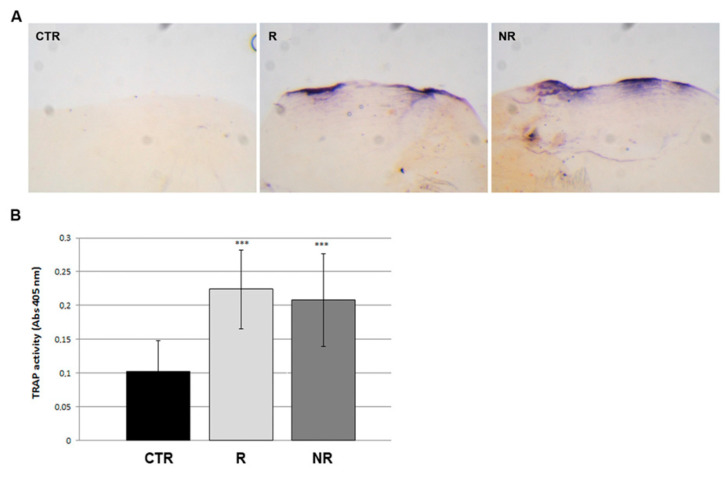
Resorption activity in scales. (**A**) Histological assay of TRAP activity in scales. Both scales from untreated fish (CTR), rotating (R) and non-rotating (NR) fish showed intense TRAP activity along the borders. (**B**) TRAP activity in scales was confirmed by biochemical assay (R vs. CTR, +117.6%, *** *p* < 0.001; NR vs. CTR, +102.3%, *** *p* < 0.001, *n* = 90 scales for each group).

To investigate the cause of bone loss phenotype, we analyzed the osteoclastic bone resorption activity by histological staining of tartrate-resistant alkaline phosphatase (TRAP), performed on whole scales from non-rotating controls versus RPM rotating fish. Scales from RPM (rotating and non-rotating) fish showed a statistically significant increase in TRAP activity along the scale border (Figure 4A) compared to the untreated control fish. The quantification of TRAP activity, performed by biochemical assay, indicated an increase of 117.6% and 102.3% in the rotating and non-rotating scales, respectively (Figure 4B), suggesting that the both microgravity simulation and vibrations induce an imbalance of bone metabolism which in turns leads to an osteoclast-dependent osteoporotic phenotype in fish scales.

### 3.4. Bone Loss Phenotype Was Not Correlated to Cortisol Production

**Figure 5:**
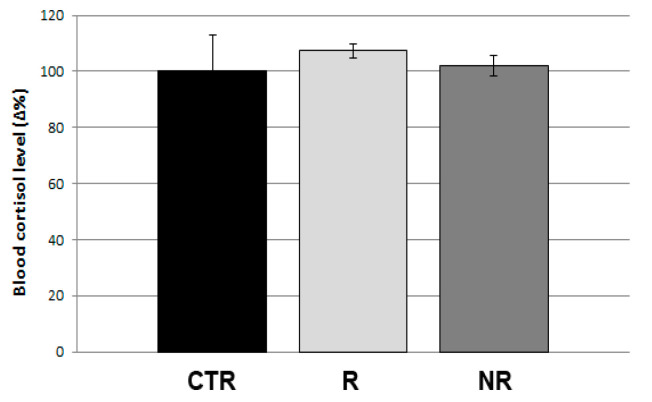
Cortisol evaluation. Cortisol level in blood of controls (CTR), rotating (R) and non-rotating (NR) fish. No modulation was highlighted (*n* = 3 fish for each group).

Since mechanical vibration can generate stress in fish, we measured the cortisol levels in the blood of non-rotating, rotating and control fish at the end of the experiment. In all the conditions, the cortisol results were unchanged, suggesting that bone resorbing activity observed in scales was not stimulated by systemic cortisol-dependent stress signals (Figure 5).

## 4. Discussion

Since space flight and gravity reduction have been associated with bone loss in astronauts, ground experiments with animal models have been projected to evaluate phylogenetic differences and the pathophysiology mechanisms. The use of fish has been proposed despite them being an animal that lives in water, an environment with different density and physical properties compared to terrestrial life [12]. Independently of the medium, animals respond to mechanical forces and, theoretically, gravity. In addition, in space colonization, animals should be carried on. The knowledge of the water animals’ behavior in different gravity conditions is relevant, as well as for ground animals [22]. Few experiments can be found in the literature concerning the modulation of fish bone tissue in microgravity, and, most of them have been performed from the view of embryonic osteogenesis [14]. However, assuming that astronauts’ osteoporosis results from remodeling of adult bone, the use of an adult animal model would be preferable. In this work, we used zebrafish to verify if the simulated microgravity modulates adult skeletal tissue in a water-living animal, as previously indicated for fish embryos. A specific water box was assembled to host adult zebrafish during the experiments and the fish scale was used to evaluate the modulation of bone metabolism after microgravity stimulation with an RPM [16].

Recently, the goldfish scale has been used in vitro as a culture system to study the bone response to hypergravity and microgravity, showing the activation of osteoclastic-specific gene expression by physical stimulation [23].

Our results obtained in vivo evidenced an osteoporotic phenotype in the scale of RPM-rotating fish as well as in non-rotating controls, suggesting that vibrations perceived by fish on the RPM basement might also modulate bone remodeling in fish scales.

Recently, the amphibious fish K. marmoratus has been used as an experimental model to investigate weight-induced musculoskeletal remodeling [24]. In this work, Turko et al. suggested that skeletal structures (gill arch) of amphibious fish had been evolutionally modified by consequence of a gravity-dependent increase in effective body weight with terrestrial acclimation. The authors deduced also that normal fish, such as D. rerio, did not develop this adaptation because they live in water in permanently simulated microgravity due to the counterbalance between gravity and the Archimedes law. In addition, the changes to the extracellular matrix evidenced in K. marmoratus were also found in mammals after gravitational loading modulation [25]. These data suggested that adult fish skeletal tissues are less responsive to microgravity than other terrestrial animal models. 

On the other hand, our data clearly indicate that the mechanical vibration alone seem to be responsible of all osteoporotic phenotype in fish scales.

It is known that vibration may cause in animals different responses at different thresholds and affects health and research results [26]. The skeletal system, a typical mechanical tissue, can be modulated in human and animals by mechanical vibrations [27]. However, some occupational medicine studies have documented a negative impact of vibrations on human health [28] whereas other studies highlighted some potential benefits of vibration, especially in skeletal tissues [29,30,31].

Taken together, the data suggest that vibration-dependent anabolic or catabolic effect on bone tissue seems to dependent on the type, frequency, target site and intensity of the vibration.

The role of both microgravity and vibration has been studied on zebrafish embryos by analyzing osteogenesis in long-term experiments [15]. The analysis of the number and shape of vertebral bodies confirmed that vibration alone is able to affect osteogenesis mechanisms. 

Vibration has been also indicated as a modulator of chondrogenic condensations in the developing zebrafish tail skeleton [32].

The Medaka scale has been used in vitro to evaluate the response to hypergravity generated by centrifugation or vibration, highlighting that the expression of rankl increases significantly after vibration loading, but not after centrifugal loading [33]. These data suggested that vibrations can unbalance bone metabolism towards resorption in zebrafish scales.

Worthy of discussion is that the space environment in which astronauts live is characterized by vibrations, such as on the ISS [34], and the possibility that vibrations together with weightlessness might contribute to space induced osteoporosis must be taken into account. 

Lastly, we considered cortisol as a metabolic modulator between mechanical stimulation and bone resorption. Cortisol, also named “stress hormone”, has been suggested to be a promoter of osteoporosis [35,36,37,38].

Nevertheless, in humans, has been demonstrated that blood cortisol levels do not increase after whole-body vibration but, on the contrary, decrease [39,40].

Our data in adult zebrafish shown that blood cortisol does not change after vibrational loading as well as in simulated microgravity, therefore we exclude cortisol as metabolic link between physical stimulation and scale bone loss.

In mammals, osteocytes modulate the remodeling of mineral tissues after mechanical stimulation [41]. In fish, some species are osteocyte-free (pike fish, medaka), whereas others possess buried cells with similar roles of osteocytes (sturgeon, zebrafish). 

However, the bone of different fishes, although lacking osteocytes, shows a similar osteon-like structure and mechanical functions and has shown osteoclast activation in internal bones after permanence in space [42]. However, zebrafish, which possess osteocytes (cellular bone), had a poorly developed osteocyte lacuno-canalicular system [43]. 

## 5. Conclusions

In conclusion, our results point out the necessity to be more cautious when using adult aquatic animal models to study weightless-linked complications than in the past. Compensative systems are always active in water-living animals. However, developing embryos are more vulnerable to mechanical stress, as demonstrated during hydrostatic pressure variations [44]. 

In addition, it would be worthwhile to set up the housing of controls when conducing simulated microgravity experiments in water-living animals, to take in account the possible effects of vibrations. This study has potential limitations. Simulated microgravity may not match with the absence of gravity found in space. Due to the available surface on the inner clinostat platform, the constrains related to microgravity simulation and the fish box volume, the systems allowed us to only test a small number of animals during the simulation.

## Data Availability

Data are contained within the article.
